# Resting State fMRI: Going Through the Motions

**DOI:** 10.3389/fnins.2019.00825

**Published:** 2019-08-13

**Authors:** Sanam Maknojia, Nathan W. Churchill, Tom A. Schweizer, S. J. Graham

**Affiliations:** ^1^Physical Sciences Platform, Sunnybrook Research Institute, Sunnybrook Health Sciences Centre, Toronto, ON, Canada; ^2^Keenan Research Centre for Biomedical Science, St. Michael’s Hospital, Toronto, ON, Canada; ^3^Division of Neurosurgery, Faculty of Medicine, University of Toronto, Toronto, ON, Canada; ^4^Institute of Biomaterials and Biomedical Engineering, Faculty of Medicine, University of Toronto, Toronto, ON, Canada; ^5^Department of Medical Biophysics, Faculty of Medicine, University of Toronto, Toronto, ON, Canada

**Keywords:** resting state fMRI, noise, motion artifacts, motion compensation, image processing

## Abstract

Resting state functional magnetic resonance imaging (rs-fMRI) has become an indispensable tool in neuroscience research. Despite this, rs-fMRI signals are easily contaminated by artifacts arising from movement of the head during data collection. The artifacts can be problematic even for motions on the millimeter scale, with complex spatiotemporal properties that can lead to substantial errors in functional connectivity estimates. Effective correction methods must be employed, therefore, to distinguish true functional networks from motion-related noise. Research over the last three decades has produced numerous correction methods, many of which must be applied in combination to achieve satisfactory data quality. Subject instruction, training, and mild restraints are helpful at the outset, but usually insufficient. Improvements come from applying multiple motion correction algorithms retrospectively after rs-fMRI data are collected, although residual artifacts can still remain in cases of elevated motion, which are especially prevalent in patient populations. Although not commonly adopted at present, “real-time” correction methods are emerging that can be combined with retrospective methods and that promise better correction and increased rs-fMRI signal sensitivity. While the search for the ideal motion correction protocol continues, rs-fMRI research will benefit from good disclosure practices, such as: (1) reporting motion-related quality control metrics to provide better comparison between studies; and (2) including motion covariates in group-level analyses to limit the extent of motion-related confounds when studying group differences.

## Introduction

Since the first report of temporal correlations between spontaneous blood oxygenation level-dependent (BOLD) signals in the bilateral motor cortices (Biswal et al., 1995), “resting-state” functional magnetic resonance imaging (rs-fMRI) has become an important tool to probe functionally connected networks throughout the brain (Smith et al., 2013b). The rs-fMRI method continues to advance the scientific understanding of brain development, aging, and disease (Woods et al., 1998; Fair et al., 2008; Supekar et al., 2009; Bettus et al., 2010; Qin et al., 2012; Lin et al., 2018), among other application areas, and affords a number of advantages over the original task-based fMRI approach for recording brain activity. For example, multiple resting-state networks can be revealed from a single rs-fMRI study without the need to administer one or more prescribed behavioral tasks, typically by measuring BOLD signal correlations relative to a “seed” region of interest, or by using multivariate component models to identify networks based on statistical criteria. The absence of the task(s) also removes the need for fMRI-compatible devices to present sensory stimuli and record behavioral responses, along with the device-related software and computer control. Thus, the relatively straightforward acquisition of the data, coupled with the wealth of information that is obtained, have spurred adoption of the rs-fMRI method for research purposes. This is especially the case for clinical neuroimaging research involving patient populations, in which the workflow of the fMRI experiment must be efficient and task performance may not be possible or is confounded by impairments related to the brain disease under study.

Although rs-fMRI is an effective tool for studying the brain function of healthy and patient populations, the measured BOLD signal fluctuations are caused not only by neuronal activity, but also by multiple other confounding factors. These include physiological effects (e.g., respiration and cardiac pulsatility) and various imperfections in MRI system hardware (e.g., heating of the imaging gradients during experiments). Of all the confounding factors, however, the effects of head motion are especially complex and troublesome. The small amplitude of BOLD signals – typically a few percent or less – ensures that millimeter-scale head motions may be problematic even after various correction algorithms are applied to fMRI data. In the case of task-based fMRI, head motion can be temporally correlated with task performance and under many circumstances, the resulting “motion artifacts” cannot be distinguished from brain activity. The interpretation of the fMRI data becomes compromised as a result (Johnstone et al., 2006). Although prescribed behavioral tasks are not a part of rs-fMRI, head motion still is problematic and may even be exacerbated when imaging individuals while they are at rest (Engelhardt et al., 2017; Huijbers et al., 2017). Numerous effects of head motion have been reported in the rs-fMRI literature. For example, sub-millimeter motions have been shown to distort functional connectivity estimates from approaches that include seed correlation analyses, graph theoretic network modularity, dual regression independent component analysis (ICA), and power spectrum methods (Power et al., 2012; Satterthwaite et al., 2012; van Dijk et al., 2012). Depending on the amplitude and spatio-temporal characteristics of the head motion, estimates of functional connectivity can be increased, decreased, or even driven to zero (Power et al., 2014). Characteristic “distance” and “orientation” dependencies of the errors have been reported in correlation-based estimates, with decreased long-distance connectivity and increased local connectivity (Power et al., 2012; van Dijk et al., 2012); and increased lateral connectivity at the expense of connectivity in the inferior–superior and anterior–posterior directions (Power et al., 2012). The effects are especially problematic in between-group studies of brain development and of neurological diseases, as the groups may differ significantly in their levels of head motion (Seto et al., 2001; Mowinckel et al., 2012; Satterthwaite et al., 2012; Haller et al., 2014). In these cases, it may be very difficult to decouple hypothesized effects (Courchesne and Pierce, 2005; Andrews-Hanna et al., 2007), from motion-related differences with the greatest effects of motion often observed in groups with the greatest brain impairment (Wylie et al., 2014).

Given these reports and the need to generate data with improved quality in the long term, this focused review discusses how head motion affects rs-fMRI data, and summarizes the existing and emerging strategies for motion correction. The pertinent characteristics of human head motion are first discussed, followed by the physical principles that cause head motion to introduce signal confounds in rs-fMRI data. The second half of the review discusses the strengths and weaknesses of various retrospective motion correction strategies, and the potential benefit that “real-time” correction techniques can provide in the future.

This focused review is not exhaustive in terms of the references that are included. Interested readers are encouraged to seek out other discourses that provide more in-depth discussion of topics that are covered here (e.g., Power et al., 2015; Esteban et al., 2019). In addition, for balance and brevity, the review focuses on the main concepts behind various motion correction strategies without explicitly mentioning and defining all their acronyms. The acronyms are available in the references that are cited.

## Head Motion: Characteristics

As a reasonable starting point, the head may be considered as a rigid body that can move in space. Three dimensional (3D) rigid body motion is usually parameterized by six degrees of freedom (DOF), for example described in Cartesian coordinates as translations in *x*- (left/right), *y*- (anterior/posterior), and *z*-axes (inferior/superior), and rotations about the *x*-axis (pitch), *y*-axis (yaw), and *z*-axis (roll). Each of the six parameters will vary as a function of time as the head moves dynamically during an rs-fMRI experiment (a time series data collection of images of the brain volume, acquired with BOLD signal contrast). In reality, the brain is not perfectly rigid, given the biomechanical properties of its constituent tissues and the pulsatile flow of blood within it (Dagli et al., 1999). Nevertheless, given the dynamics of the motions involved and the millimeter spatial resolution that is presently available on most MRI systems operating at 1.5 and 3.0 T, the rigid body approximation is very reasonable. The rapid imaging protocols that are used in rs-fMRI [typically echo planar imaging (EPI) or spiral k-space readouts] also ensure that motion is effectively “frozen” during the time needed to encode the spatial information for each image slice (∼50 ms or less) in a typical two-dimensional (2D) multi-slice imaging protocol. Although each slice samples the head motion at a slightly different point in time, this issue is usually dealt with effectively by temporal interpolation of slices to a single time point (Parker et al., 2017).

Although head motion often varies considerably from subject to subject, multiple studies have revealed that certain general characteristics are common. In healthy individuals, for example, translations in the inferior/superior direction together with a “nodding” rotational motion are often evident, possibly with superposition of more rapid oscillatory motion from the respiratory cycle (Seto et al., 2001). This pattern of motion arises because a pivot point occurs at the back of the head or the base of the neck while the subject lies supine in the magnet bore, with relatively constrained motion in the other directions. This common pattern has implications for the extent of motion in different brain regions: anterior frontal and orbitofrontal areas are likely to be more affected than posterior areas such as the primary visual cortex. Furthermore, this motion is not well represented by fluctuations in just one DOF in Cartesian coordinates – instead, coupled translation and rotation signals are observed that may be difficult to resolve unambiguously.

Another characteristic feature of head motion is that the temporal patterns of movement and associated artifacts do not display band-limited frequency content. As such, frequency filtering commonly applied in rs-fMRI to isolate the frequency range of interest (∼0.01–0.1 Hz) may be ineffective for motion correction, and can even smear motion contamination across the entire dataset if not applied carefully (Carp, 2013). Low-frequency, autocorrelated trends are readily apparent in rs-fMRI data due to motion, and work initially focused on developing methods other than frequency filters to remove these artifacts while retaining the true fMRI signal content (Woods et al., 1998; Lund et al., 2006). More recent work has focused on the need for specialized methods to account for transient motions (Satterthwaite et al., 2013), for example due to involuntary twitches or tics, which also occur at non-trivial levels.

There is also evidence that head motion can differ across various populations of subjects. Task-based fMRI studies show that patient populations, older adults, and pediatric subjects exhibit larger motions compared to young healthy adults (Seto et al., 2001; Yuan et al., 2009; Haller et al., 2014; Graham et al., 2016; Huijbers et al., 2017). For example, patients with stroke, Alzheimer’s Disease, bipolar disorder and schizophrenia move more compared to age-matched healthy subjects (Seto et al., 2001; Haller et al., 2014; Huijbers et al., 2017). Similarly, young children and older adults show larger motions when compared to young adults (Seto et al., 2001; Yuan et al., 2009). Elderly subjects show more random head motions whereas young adults move more slowly and rhythmically (Graham et al., 2016). Sex-related differences have also been observed, with girls showing less tendency to move than boys during three of four language tasks in a task-based fMRI study (Yuan et al., 2009). Finally, less engaging task paradigms and rs-fMRI protocols may also lead to levels of head motion that are higher than those observed in task-related fMRI measurements (Huijbers et al., 2017) although more research would be useful in this area. As the amount of rs-fMRI data increases and becomes more freely accessible throughout the human brain mapping community, the opportunity should be taken to evaluate the head motion characteristics in studies with large sample size and different subject populations, as this may help to inform motion correction and data analysis methods in the future.

## Head Motion Artifacts

The consequences of head motion on rs-fMRI data can be very complex. Rather than producing a single type of image artifact, multiple types are possible with very different physical mechanisms. A list of the possibilities is given below. This list is not exhaustive, and some of the possibilities are more commonly appreciated than others.

### Partial Volume Effects

Functional MRI data are almost always acquired within the static coordinate frame of the MRI system, assuming that each voxel represents the signal content of the same brain structure for the entire duration of the time series data collection. However, head motion causes the proportion of various brain tissue types in a voxel to fluctuate over this duration, each with slightly different MRI signal contrast properties (Stanisz et al., 2005). This is commonly referred to as the “partial volume effect” (Hajnal et al., 1994) and is most problematic for voxels in the vicinity of tissue boundaries where large signal differences occur [e.g., between gray matter (GM) and white matter (WM), and especially between GM and cerebrospinal fluid (CSF)]. The partial volume artifact characteristically appears as spurious correlated signal fluctuations that rim the surface of the brain, or that occur along the interhemispheric fissure. It is increasingly realized that as fMRI protocols are developed with greater spatial resolution, for example using ultra-high field systems at 7 T or beyond, the reduction of voxel size will cause the partial volume effect to increase (Zaitsev et al., 2017) and thus better correction strategies will be needed (see section “Correction Strategies” below).

### Spin History Effects

As mentioned above, head motion tends to have major components that involve “nodding” and displacements in the inferior–superior direction (Seto et al., 2001). As fMRI protocols commonly adopt 2D multi-slice imaging with an axial or oblique-axial slice prescription, brain tissue will inevitably move through each slice, producing an artifact that is usually referred to as the “spin history effect.” In an rs-fMRI experiment, the baseline signal intensity is a function of multiple MR acquisition parameters and MR tissue properties, but the quantities relevant to spin history are the flip angle (θ) of radiofrequency excitation, the repetition time (TR) determining the temporal resolution of the rs-fMRI time series, and the longitudinal relaxation time (T1) at a particular voxel location. At the start of any time series data acquisition, it takes several TR intervals to establish the steady-state baseline signal intensity, which is achieved from a balance of how far the tissue magnetization or “spins” are flipped toward the transverse plane, and the time allotted for T1 recovery before the next θ pulse is applied. Ideally, the θ value should be constant through the slice, but in reality there is significant spatial non-uniformity. Thus, through-plane motion disturbs the steady state magnetization of the imaged slice by introducing spins with different excitation history. The steady state will also be disturbed if tissues with different T1 values move in and out of the slice – which is particularly observable for voxels that include blood vessels.

Spin history effects have been modeled empirically (Friston et al., 1996; Muresan et al., 2002) and in phantom experiments to establish the dependency on MR acquisition parameters and tissue properties (Yancey et al., 2011). The characteristic behavior is that a discrete through-plane displacement causes a signal transient that may be similar in amplitude to the rs-fMRI signal and requires several TR intervals to attenuate. In cases of slow, smooth motion, spin-history artifacts may be quite difficult to distinguish from the true BOLD fluctuations in rs-fMRI data.

### Dynamic Geometric Distortions

Although EPI and spiral k-space readouts provide good temporal resolution for rs-fMRI experiments, both are very sensitive to spatial non-uniformity in the static magnetic field (Jezzard and Clare, 1999; Glover, 2012). Automatic “shimming” procedures are available on all clinical MRI systems and provide some benefit, but the differences in magnetic susceptibility at interfaces between brain tissues, bone, and air are sufficiently large that regions of geometric distortion and signal loss remain – typically in inferior frontal and inferior lateral temporal areas (Ojemann et al., 1997). It is well appreciated that a constant correction for these effects may be needed at each point in the rs-fMRI the time series data collection, but dynamic corrections may be needed as well (Zaitsev et al., 2017). Lung ventilation effects during the respiratory cycle cause magnetic field fluctuations in inferior brain regions at 3 T and above (Raj et al., 2001; Van de Moortele et al., 2002). Furthermore, head motion causes the susceptibility-induced field non-uniformities to fluctuate in a manner such that the boundary conditions at each tissue interface satisfy Maxwell’s Equations. The end result is dynamic geometric distortions that are observable in the EPI phase-encoding direction (Wu et al., 1997; Jezzard and Clare, 1999; Andersson et al., 2001). The effects are non-linear with respect to motion estimates and vary depending on the position and orientation of the tissue interfaces relative to the main magnetic field, the amount of head motion, and the magnetic field strength.

### Coil Sensitivity

Multi-channel receiver coils are now an established part of fMRI protocols, providing higher signal-to-noise ratio (SNR) than previously achievable and enabling higher temporal resolution through various parallel imaging reconstruction approaches (Pruessmann, 2006). Channel count continues to increase, with 64-channel coils currently available from at least one major MRI system vendor. The higher the channel count, the smaller each individual element becomes. The associated area of sensitivity of each element also becomes more localized, with steeper spatial sensitivity gradients. This implies that at some point, multi-channel receiver coils will become appreciably sensitive to head motion, if the translation or rotation of brain tissue becomes sufficiently large in relation to the sensitivity gradients of the individual coil elements. Two recent reports have indicated that this problem may be relevant for rs-fMRI at 3 T in a 16-channel coil geometry, for a conventional EPI k-space readout (Faraji-Dana et al., 2016a) as well as for parallel imaging reconstruction, with worse artifacts occurring as the acceleration factor was increased (Faraji-Dana et al., 2016b). In both cases, it was possible to suppress these artifacts by tracking and correcting for the relative motion between the head and the receiver coil, at each point during the fMRI-time series data collection.

## Correction Strategies

Given the complexity of the problem, it is not surprising that a multifaceted approach is needed in the quest to achieve full and robust motion correction in rs-fMRI data. A brief summary of the available correction strategies is given below. The choices range from simple commonsense approaches, to more sophisticated retrospective corrections as well as “real-time” corrections.

### Head Restraints and Behavioral Intervention

At the outset, it would seem straightforward simply to restrain individuals so that no head motion occurs during rs-fMRI. The problem would thus be solved at the source, without introducing artifacts into the data. Unfortunately, it is often very difficult to achieve this goal in practice. Mild head restraint is an essential part of all fMRI procedures: padding between the head and the coil is commonly adopted (with other options available such as the use of vacuum pillows, and thermoplastic facial masks fixed to the MRI table), whereas bite bars and even more restrictive clamping systems are used less frequently (Bettinardi et al., 1991; Green et al., 1994; Righini et al., 1996; Schültke et al., 2013). Although restraints decrease the extent of head motion in cooperative subjects, in many cases the milder forms of restraint are ineffective at eliminating some component of motion at the sub-millimeter and millimeter level, such as nodding. However, the stronger restraints have the potential to increase claustrophobia, can become uncomfortable and tiresome especially for lengthy fMRI sessions, and in some cases can exacerbate motion as subjects try to alleviate associated pain or pressure (Zeffiro, 1996). Brain activity is also likely to be altered as a result, especially in very young or very old healthy individuals. Furthermore, clinical contraindications make strong restraints unacceptable for certain patient populations (Zeffiro, 1996; Schültke et al., 2013).

Subjects are also commonly instructed to “try to lie still and not move” as part of setup and positioning prior to rs-fMRI experiments. For these instructions to have the intended effect, subjects must appreciate the small level of motion that can be tolerated and also must remain vigilant at keeping still. As mentioned above, pediatric and patient populations may not be able to fulfill these requirements, with reduced rs-fMRI data quality as a consequence. For example, children are more prone to head motion when tasks are less engaging, making motion correction strategies important for rs-fMRI acquisitions (Yuan et al., 2009; Engelhardt et al., 2017). Pre-training using “mock scanning” or “fMRI simulator” sessions may help to reduce the need for sedation when imaging children and may provide more runs with usable MRI data (Epstein et al., 2007; De Bie et al., 2010; Barnea-Goraly et al., 2014), but significant benefit of this approach is not consistently demonstrated (Thieba et al., 2018; Li et al., 2019). Training tools and interventions such as watching a movie and/or motion feedback training (visual or verbal) have shown promise in children, young adults and stroke patients (Vanderwal et al., 2015; Graham et al., 2016; Greene et al., 2018). In the case of the movie paradigm, however, functional connectivity measures are contaminated by brain activity associated with watching the movie and cannot be considered truly “resting-state.” Collectively, these methods require additional set-up, lengthen the duration of the imaging session, and are not widely adopted yet, at least partly for these reasons. Another alternative is to monitor head motion and adjust the length of the time series data acquisition so that enough data of sufficient quality are collected (Dosenbach et al., 2017). Although useful, this approach is rather open-ended and may be inefficient for patients with moderate-to-excessive motion.

### Imaging Protocol

The rapid 2D multi-slice imaging methods commonly used in rs-fMRI not only provide adequate temporal resolution to sample BOLD responses, but also afford some protection against motion artifacts. In addition to the “snap-shot” imaging capability provided by the raster scan k-space readouts used in EPI, the spiral k-space readout intrinsically compensates for motion in the plane of each image slice (Glover and Lai, 1998). Researchers have also continued to develop imaging methods with even better motion compensation (Lee et al., 2010; Krämer et al., 2012; Graedel et al., 2017; Kecskemeti et al., 2018). The increasingly popular alternative involves simultaneous multi-slice acquisitions together with parallel imaging reconstruction to provide increased temporal resolution, better snap-shot imaging capability, and robustness to static and dynamic geometric distortion (Feinberg et al., 2010; Setsompop et al., 2012; Zahneisen et al., 2014b). However, this approach introduces a different set of noise characteristics which may have implications for rs-fMRI analysis (Golestani et al., 2018). Dual- and multi-echo imaging methods have also been receiving attention recently because the acquisition of two or more images of each slice at different echo time (TE) values helps to isolate BOLD signals from noise. This can be achieved by regressing low TE value data (with minimal BOLD weighting plus noise) from higher TE value data (with more optimal BOLD weighting plus noise) (Buur et al., 2009; Bright and Murphy, 2013), or by a more complex multivariate denoising approach relying on signal decay properties (Kundu et al., 2013). Dual- and multi-echo approaches must be applied judiciously, however, so that the spatiotemporal resolution of 2D multi-slice rs-fMRI is not compromised.

### Retrospective Motion Correction

Over the years, many strategies have been developed that help to suppress the effects of head motion after fMRI data have been collected. These “retrospective” methods are an essential part of processing rs-fMRI signals and are easily implemented as part of freeware analysis packages developed and applied by the functional neuroimaging research community (e.g., Esteban et al., 2019).

#### Rigid-Body Registration

Volumetric rigid-body registration primarily corrects for partial volume effects and is typically viewed as an essential step of rs-fMRI analysis. Head motion parameters are estimated iteratively with six DOF by optimizing a cost function that quantifies the similarity between each image in the time series and a reference image (Friston et al., 1995; Cox, 1996; Jenkinson et al., 2002; Oakes et al., 2005). The reference image should be chosen carefully (such as the average image over the time series), as the error in motion parameter estimates increases with the extent that each image must be re-aligned. Although very useful, volumetric rigid-body registration does have some limitations. Most implementations do not correct for motion that occurs during multi-slice acquisition of the entire brain volume, so slice-to-volume as well as slice-to-slice registration approaches have been developed (Kim et al., 1999, 2008; Yeo et al., 2008; Beall and Lowe, 2014; Chen et al., 2015; Ferrante and Paragios, 2017). The accuracy of motion estimates also depends on the signal quality in the image slices, which are acquired at low spatial resolution and at relatively low SNR, with BOLD-related signal variations that can bias motion estimates toward neural activations depending on the choice of the cost function (Freire and Mangin, 2001). The latter effect can be mitigated in principle by simultaneously optimizing the registration while estimating fMRI signals, although the approach has only been tested for task-based fMRI thus far (Orchard et al., 2003). Furthermore, the registration process inherently requires resampling and interpolation so that all motion-corrected images utilize a common Cartesian coordinate system. This can further reduce spatial resolution and bias activation estimates (Grootoonk et al., 2000; Yuan et al., 2016). Lastly, volumetric registration algorithms work well for small head movements, but become less accurate or fail completely for larger motion (Oakes et al., 2005; Morgan et al., 2007). In particular, large motions can invalidate the assumption of rigid-body motion as a consequence of the geometric distortions introduced by dynamic magnetic field inhomogeneity (Elliott et al., 2004). In such cases, complex affine or non-linear transformation models are beneficial, as well as use of dynamic maps of the magnetic field (Hutton et al., 2002; Roopchansingh et al., 2003; Sutton et al., 2004; Visser et al., 2012; Ooi et al., 2013b), although these methods are more computationally intensive and have not been widely adopted yet.

#### Linear Regression

Various linear regression strategies are also commonly adopted to address the residual motion-related signal variance that can arise from imperfect volumetric rigid-body registration. For example, the six time-dependent motion parameter estimates that are output from the registration are easily applied in multiple linear regression to remove these “nuisance” effects from the rs-fMRI data. The approach has been extended further to 12 parameters (including temporal derivatives; Power et al., 2012), 24 parameters (squares of the motion parameters and temporal derivatives; Friston et al., 1996; Satterthwaite et al., 2013; Yan et al., 2013) and even 36 parameters (squares of the motion parameters, and both first and second temporal derivatives; Power et al., 2014). Specialized regression procedures have also been proposed for group comparisons (Satterthwaite et al., 2012; Yan et al., 2013). The use of higher-order regressors has demonstrated greater reduction in motion-related variance (Lund et al., 2005) and has been suggested for high-motion subjects (Satterthwaite et al., 2013; Yan et al., 2013; Yuan et al., 2016), for which low-order regression (6 or 12 parameters) has been found less effective (Power et al., 2012; Satterthwaite et al., 2013). However, concerns associated with overfitting and removal of BOLD signals arise in cases where head motion is minimal and large numbers of nuisance regressors are used. Direct evidence of this effect has been shown in task-based fMRI (Johnstone et al., 2006; Ollinger et al., 2009) whereas more investigations remain to be undertaken in rs-fMRI. Moreover, motion parameter estimates are often highly coupled and fitting with better statistical power is achieved when a method such as principal component analysis (PCA) is used to reduce the dimensionality of the nuisance regressors (Woods et al., 1998).

When considering regression approaches, it should also be recognized that fMRI signal changes from movements can have a latency of several seconds (due to spin history effects, for example) (Power et al., 2014). Simple motion parameter regression cannot completely remove such deviations and thus more sophisticated methods are of interest, such as the use of more nuisance regressors as indicated above. Another approach considers that BOLD signals arise predominantly from GM, and thus additional effects from motion and non-neural sources can be removed by using spatially averaged time series signals of WM and CSF (WM-CSF) as nuisance regressors, and possibly the related derivatives (Weissenbacher et al., 2009). To address the dimensionality concerns raised above, a regressor from WM-CSF PCA space can be used (Behzadi et al., 2007; Muschelli et al., 2014). Different WM regressors can also be obtained for each GM voxel, accounting for spatial variations in WM noise that may not be apparent in the average regressor (Jo et al., 2010). Both the latter methods have been shown to perform better than regression of the average WM-CSF time series. Irrespective of how the WM-CSF regressors are derived, however, they should be implemented with “erosion” of the corresponding spatial masks to avoid contamination from adjacent GM voxels – otherwise the rs-fMRI signal can be attenuated (Jo et al., 2010). Furthermore, when applying WM regressors, it should be recognized that they may represent signal of functional origin (Ding et al., 2013; Peer et al., 2017). More research on this topic will be important in clarifying the noise or information characteristics of WM signals.

An additional nuisance regressor of potential interest is obtained by spatially averaging the rs-fMRI time series data over the whole brain. This “global signal” is usually correlated with the first PC of the whole brain time series (Carbonell et al., 2011). The value of global signal regression (GSR) is currently in dispute (Murphy and Fox, 2017; Xu et al., 2018). Originally, GSR was performed assuming that any source that modulates the global brain signal is non-neural (Desjardins et al., 2001), but more recent studies have shown that the global signal does contain measurable neural contributions (Schölvinck et al., 2010; Wong et al., 2016) and even distinguishes healthy subjects from schizophrenia patients (Hahamy et al., 2014). Nevertheless, many studies have demonstrated the usefulness of GSR for mitigating motion-related noise, although with residual artifacts that depend on the distance between functional connections (Yan et al., 2013; Power et al., 2014; Ciric et al., 2017). Other studies report that GSR introduces false anticorrelations (Murphy et al., 2009; Weissenbacher et al., 2009). This discrepancy in the literature may relate to the level of non-neural noise that has a global effect on the rs-fMRI signal, and suggests that it may be useful to quantify the global noise level to determine whether GSR should be adopted (Chen et al., 2012).

#### Scrubbing

Involuntary head motion can produce substantial transients in the rs-fMRI signal. The transients can be identified by establishing a threshold for outlier signals, for example based on relative signal difference followed by corrections such as “spike” regression (Lemieux et al., 2007), or scrubbing/censoring (ignoring) the erroneous data (Power et al., 2012). Both methods are effective at removing transient motion artifacts (Satterthwaite et al., 2013; Power et al., 2014; Ciric et al., 2017; Parkes et al., 2018), with some notable caveats in the latter case. Temporal interpolation or spectral decomposition of un-scrubbed data can be used when outliers occur at multiple adjacent time points, but this must be done carefully to avoid residual artifacts and subtle motion bias (Power et al., 2014). Moreover, rs-fMRI analysis can be complicated by the variation in temporal DOF across subjects or groups of subjects with considerable differences in head motion (Parkes et al., 2018). Data sets with a greater number of scrubbed spikes will have systematically reduced temporal autocorrelation. “Trimming” each dataset to equal length provides a simple solution, although the reliability of functional connectivity estimates may be reduced (Birn et al., 2013; Power et al., 2014). Subjects with high levels of motion may need to be excluded if many points in the rs-fMRI time series are scrubbed.

#### Data-Driven Methods

Various multivariate methods are useful to determine what components, or “features,” exist in the rs-fMRI data without imposing a mathematical model *a priori* for the signal and noise properties. Such data-driven methods are advantageous because they place less burden on the operator to identify all types of motion artifacts and implement specific correction methods – potentially allowing results to be replicated more easily across studies. However, data-driven methods do require some form of *post hoc* feature selection of the components (and the number of components used) to identify the signals of interest and remove structured noise. For example, mutually orthogonal features are identified by PCA, which has been used to remove motion-related signal fluctuations at the edge of the brain for improved temporal SNR compared to use of motion parameter regression (Patriat et al., 2015). In addition, ICA (Thomas et al., 2002) is popular to identify features based on statistical independence rather than orthogonality. Manual identification of noise-related ICs requires detailed knowledge of the spatiotemporal properties of the rs-fMRI signal (see Griffanti et al., 2017 for guidance) and is laborious and operator-dependent, but multiple automatic methods have been developed that are robust and objective (Tohka et al., 2008). These include methods specifically focused on removing physiological noise associated with cardiac pulsatility and respiration (Beall and Lowe, 2007; Perlbarg et al., 2007), and more general artifact removal methods with different processes for feature selection (Bhaganagarapu et al., 2013; Salimi-Khorshidi et al., 2014; Pruim et al., 2015b). Work has also been done to compare the effectiveness of these methods, as well as in relation to other de-noising approaches such as spike regression and scrubbing (Pruim et al., 2015a; Parkes et al., 2018). Additional comparisons of this type will be necessary to establish whether one or more methods are particularly advantageous across different populations of test subjects in rs-fMRI studies.

#### Other Methods and Considerations

Briefly, it is important to make three additional comments about retrospective correction of motion artifacts in rs-fMRI data. First, comparative work on volumetric versus surface-based fMRI analysis shows that the latter provides superior inter-subject alignment and better preservation of functional regions upon smoothing (Anticevic et al., 2008; Tucholka et al., 2012; Smith et al., 2013a). Even so, retrospective motion correction is usually performed as a preliminary step in the volumetric domain prior to the projection of de-noised fMRI data onto the brain surface. Second, artifact reduction is an intensive field of MRI research and new correction methods are continuously being developed, some of which may have significant merit without aligning to the categories listed above. One example is a method called “wavelet despike” that has been developed to identify dynamic events occurring across various frequencies, for the removal of sudden spikes from head motion as well as slower spin-history related artifacts (Patel et al., 2014). This method is particularly useful for subjects with elevated head motion and is capable of reducing or even removing distance-dependent connectivity artifacts without the need for scrubbing (Patel et al., 2014). Third, it is evident that because no gold-standard protocol exists to correct artifacts in rs-fMRI data, the data analyst is confronted with choosing from very many rs-fMRI artifact correction methods, many of which have multiple parameter settings. Multiple correction methods must be selected to suppress artifacts most successfully, and the various methods are likely to interact with one another, sometimes in an order-dependent fashion. This state of affairs has led to multiple studies that compare various correction methods and/or their interaction effects, using various metrics to indicate the quality of the rs-fMRI results (Churchill et al., 2012a, b; Carp, 2013; Hallquist et al., 2013; Satterthwaite et al., 2013; Power et al., 2014; Pruim et al., 2015a; Shirer et al., 2015; Ciric et al., 2017; Vytvarová et al., 2017; Gargouri et al., 2018; Parkes et al., 2018). Such work will continue to be necessary as MRI systems, imaging protocols, and methods of analysis improve over time.

### Real-Time Motion Correction

Although patient setup procedures, use of rapid imaging acquisitions, and retrospective de-noising approaches are commonly adopted in rs-fMRI experiments, another class of correction methods described as “real-time,” “adaptive,” or “prospective” show considerable promise and may become essential tools in the long term. Here, the term “real-time” is adopted for these methods, which depart from typical rs-fMRI protocols that produce reconstructed images in a Cartesian coordinate system that is static with respect to the MRI system. Instead, images acquired with real-time motion correction are reconstructed in a moving coordinate system that is fixed to the head. In principle, images viewed in the moving coordinate system will appear to be static, provided that rigid body motion is a good approximation. (In reality, effects that violate this assumption will also have to be corrected either in real-time or retrospectively, as indicated below). Real-time motion correction requires (a) a method to track head motion, usually relative to an initial head position and orientation; and (b) incorporation of the tracking data to update MRI spatial encoding synchronously with the moving coordinate system. The latter requirement necessitates software modifications to the underlying image acquisition method (e.g., EPI). Depending on how rapidly and accurately the update occurs, real-time approaches have the potential to account for both partial volume effects and spin-history effects in very convenient fashion. In cases where the real-time update is relatively slow, prospective correction can be added to account for the lag between motion measurement and acquisition of the next multi-slice image dataset – using a Kalman filter, for example (White et al., 2010). Various real-time motion correction methods exist, categorized below based on the choice of motion tracking strategy.

#### Navigator Echoes

Magnetic resonance signals that are acquired and spatially encoded specifically for position tracking are known as “navigator echoes” and were among the first methods of real-time motion correction developed for fMRI (Lee et al., 1996, 1998). The main advantage of such methods is that position tracking is achieved without requiring custom ancillary hardware or fiducial markers (see below). Navigator echoes have progressed from tracking motion in 1D (Ehman and Felmlee, 1989) to full 3D capability (Welch et al., 2002; Wastiaux et al., 2006; Tisdall et al., 2012) based on calculations performed in k-space (Lin et al., 2010) or image space (White et al., 2010; Hoinkiss and Porter, 2017). However, the methods have not been widely adopted in fMRI studies to date (Boksman et al., 2005). Possible reasons for this include (a) insufficient position tracking accuracy for fMRI applications, arising from sensitivity to imperfections such as gradient non-linearity and magnetic field inhomogeneity; and (b) potential disruption of the steady state magnetization in brain regions where functional connectivity is of interest.

#### Image-Based Methods

A more popular method for real-time motion correction involves the use of volumetric image registration to track the change in head position and orientation at each point in the fMRI time series in relation to a reference volume of multi-slice images (Thesen et al., 2000). This approach is now a standard option on some MRI systems, and assumes that multiple effects are negligible: head motion on the timescale of the TR interval (typically ∼2 s); dynamic geometric distortion; and other artifacts that violate the rigid-body assumption, such as interactions between head motion and coil sensitivity. One or more of these assumptions may not always be valid. For improved functionality, a revised version of this method has recently been implemented to take advantage of simultaneous multi-slice fMRI for higher temporal resolution and intra-volume motion correction (Hoinkiss et al., 2018).

#### Other Position Tracking Devices

Many additional methods have been investigated for real-time motion correction that either adopt novel MRI signal approaches for position tracking, or other MRI-compatible sensor technologies. “Active marker” methods use at least three non-collinear RF micro-coils, each containing an MRI-sensitive material, as fiducials to measure rigid-body head motion with minimal impact on temporal resolution (Erhart et al., 1998; Krueger et al., 2006; Ooi et al., 2009, 2013a). “Passive marker” approaches have also been explored that use small pickup coils for position tracking based on the voltages induced by imaging gradients (Haeberlin et al., 2014; Aranovitch et al., 2018). As for navigator echoes and image-based methods, active and passive MRI marker devices can also suffer from instrumental imperfections that introduce errors in signal localization. Nonetheless, improved image stability has been demonstrated in standard EPI sequences (Ooi et al., 2011) as well as increased statistical significance for fMRI (Muraskin et al., 2013). The most recent and sophisticated work in this area uses an inductively coupled microcoil and a series of other passive marker components: a pickup coil, magnetometer, accelerometer and angular rate sensor. When all the sensor measurements are combined, position tracking with sub-millimeter accuracy is achievable from a single fiducial device (van Niekerk et al., 2019).

Optical sensors are also attractive for their high temporal resolution and spatial accuracy, and intrinsic MRI-compatibility. The original work involved laser interferometry (Eviatar et al., 1999), but was not pursued due to impracticalities in achieving line-of-sight and mirror adjustment. Better results are achieved using one or more optical cameras to track reflective fiducial markers affixed to the head (Zaitsev et al., 2006; Maclaren et al., 2012; Todd et al., 2015). These methods enable a tracking accuracy of ∼5–100 μm with temporal resolution of ∼20–50 ms, exceeding the capabilities of most MRI-based methods (Eschelbach et al., 2018). However, there are also some concerns about the practicality, cost and robustness of these methods at present. Calibration is required to transform optical position tracking data into the spatial coordinates of the MRI system, which may be time-consuming (Maclaren et al., 2018). Calibration errors can create further artifacts (Zahneisen et al., 2014a) that must be corrected retrospectively (Aksoy et al., 2012). The cost of optical tracking systems tends to be high, due to hardware considerations involving the MRI-compatibility of the cameras, and the research and development required to develop motion-correction capabilities with good calibration and real-time integration in MRI systems and imaging protocols. The camera view of markers (typically through openings in the head coil) may be obstructed if motion is substantial, and there is the general concern with all fiducial marker approaches (optical and other) that movement of the skin, for example due to frowning or facial expressions, may not accurately reflect motion of the brain. Each of these problems is being actively investigated and ameliorated (Singh et al., 2015; Benjaminsen et al., 2016; Eschelbach et al., 2017; Frost et al., 2018). Notably, optical motion correction has been shown to improve temporal SNR of both resting state and task-based 3D EPI acquisitions (Todd et al., 2015), with demonstrated benefits for increased significance and sensitivity of connectivity measures (Chu et al., 2018). Based on the promising outcomes of this collective work, optical tracking devices are also available for MRI applications commercially through third-party vendors, and are starting to be offered by MRI system vendors themselves.

One final comment is required about real-time motion correction methods for rs-fMRI. The existing literature in this area predominantly relies on the assumption of rigid-body head motion and, as emphasized earlier, this is likely insufficient for full suppression of motion artifacts. For example, residual geometric distortions will likely be present due to motion-induced dynamic magnetic field inhomogeneities, which can be resolved by real-time shim updates or by distortion corrections from time-dependent field maps (Ooi et al., 2013b; Rotenberg et al., 2013). Corrections for the interaction between head motion and multi-channel coil sensitivity can also be included (Faraji-Dana et al., 2016a, b). More research is needed to establish what combinations of retrospective and real-time corrections are most appropriate for rs-fMRI analyses, with the promise of more robust methodology and improved detection sensitivity in the future.

## Conclusion

Despite its utility in neuroscience, rs-fMRI is confounded by the effects of head motion during data collection, which may result in complex spatial-temporal patterns of artifact. Diverse and efficacious methods are now available that can be combined to correct for these artifacts. Much progress has been made to improve rs-fMRI data quality, but the existing methods are not yet sufficiently robust to provide full control for motion-related confounds. Real-time correction methods show considerable promise toward reaching this goal in the future. At present, however, the following recommendations represent our view of how to address the potential for confounds in rs-fMRI experiments due to motion artifacts – reasonably, and transparently. Neuroimaging data analysts should:

• report summary statistics of the head motion characteristics for the group(s) under study, including whether group differences in head motion are statistically significant;
• report and justify the methods used in the research to correct for motion artifact;
• include statistical corrections in group level comparisons to ensure that, as much as is reasonably possible, motion artifacts to do not introduce confounds in the interpretation of rs-fMRI results; and
• survey the fMRI literature for ongoing improvements in motion artifact correction methods, and evaluate and incorporate new methods as appropriate to maintain state-of-the-art capabilities.

These practices will help to advance the neuroscientific research that can be conducted using rs-fMRI, as will the continued focus on technical developments to ensure that motion artifacts become less of a problem in rs-fMRI data.

## Author Contributions

SM and SG were responsible for drafting and editing of the manuscript. NC and TS provided the additional edits for this manuscript.

## Conflict of Interest Statement

The authors declare that the research was conducted in the absence of any commercial or financial relationships that could be construed as a potential conflict of interest.
